# Delivering insecticide-treated nets (ITNs) through a digitized single-phase door-to-door strategy: lessons from Ondo state, Nigeria

**DOI:** 10.1186/s12936-024-05145-0

**Published:** 2024-10-28

**Authors:** Laitan Adeniyi, Elisabeth G. Chestnutt, Kunle Rotimi, Azuka Iwegbu, Olusola Oresanya, Julianna Smith, Kolawole Maxwell, Tarekegn A. Abeku

**Affiliations:** 1 Malaria Consortium, Abuja, Nigeria; 2 Malaria Consortium, London, UK; 3 London School of Hygiene & Tropical Medicine, London, UK

**Keywords:** Malaria, Malaria prevention, Insecticide-treated nets, Vector control, Nigeria, COVID-19

## Abstract

**Background:**

The use of insecticide-treated nets (ITNs) is a strategy recommended by the World Health Organization (WHO) for malaria prevention. In Nigeria, ITNs have been periodically distributed since 2007 through campaigns. Campaign activities and assets are typically tracked using either a paper-based or digital system. In 2017, a digital approach was introduced in Ondo state for tracking attendance at training sessions as part of the ITN campaign. Following the success of the 2017 introduction, subsequent campaigns planned to digitise other aspects of the campaign to improve accountability and efficiency of the ITN distribution. The COVID-19 pandemic posed additional challenges for the ITN distribution planned for 2021 and adaptations were made to the programme strategy to ensure the campaign could go ahead safely. This article presents lessons and experiences from the 2021 ITN distribution campaign in Ondo state, Nigeria.

**Methods:**

The campaign used RedRose, a customised mobile application, to monitor the planning and delivery of the campaign, collect household information including training personnel and tracking the transfer of ITNs between distribution hubs and households. ITNs were delivered through a single-phase door-to-door distribution strategy.

**Results:**

The campaign distributed 2,965,125 ITNs covering 1,057,577 households across Ondo state. The digital application was beneficial for monitoring the quality of implementation and tracking assets and staff to ensure safety.. The single-phase door-to-door approach was more convenient for households compared to fixed-point distribution but increased the workload for mobilization and distribution teams.

**Conclusions:**

Single phase door-to-door strategy using digital tools was an effective method to increase coverage of ITNs while closely tracking the progress of distribution campaigns. High-quality population data are needed to further improve the planning and implementation of ITN campaigns and other health interventions.

## Background

Insecticide-treated nets (ITNs) have been a core component of malaria control and elimination strategies for almost two decades, averting an estimated 450 million cases between 2000 and 2015 [1, 2]. The World Health Organization (WHO) guidelines state that households should have one ITN for every two people and estimates the life span of an ITN to be three years, although net retention times may be much lower [1]. The WHO recommends that mass distribution campaigns are carried out periodically to replace ITNs, and complemented by continuous distribution through locally appropriate channels, such as antenatal care clinics and the expanded programme on immunization [3].

The use of digital platforms for data collection have shown significant benefits when compared to paper-based systems, improving the accuracy of reporting and allowing real-time monitoring and data use for decision-making [4–6]. In 2017, a digital platform was piloted in Kwara state to track the distribution of ITNs. The same year Ondo state used a digital platform during the ITN distribution campaign to track attendance at training sessions [7]. Following the success of these campaigns, Ondo state planned to digitise the ITN distribution with its cash and asset transfer processes during the 2021 ITN campaign.

The emergence of a novel coronavirus in 2019 and the subsequent COVID-19 pandemic impacted the 2021 ITN campaign. To ensure the campaign could go ahead safely, several adaptations were made to the campaign, in particular to the distribution strategy. During the 2021 campaign a single-phase door-to-door distribution strategy was used to reduce inter-household mixing. Both the household registration and net distribution steps were completed in one phase rather than the previous two-phase process which required registration followed by net distribution at fixed points where household representatives assembled for receiving nets in exchange of net cards issued during registration. This paper details the lessons from digitising the ITN distribution campaign and the adaptations made to the distribution strategy.

## Methods

### Context

**Fig. 1:**
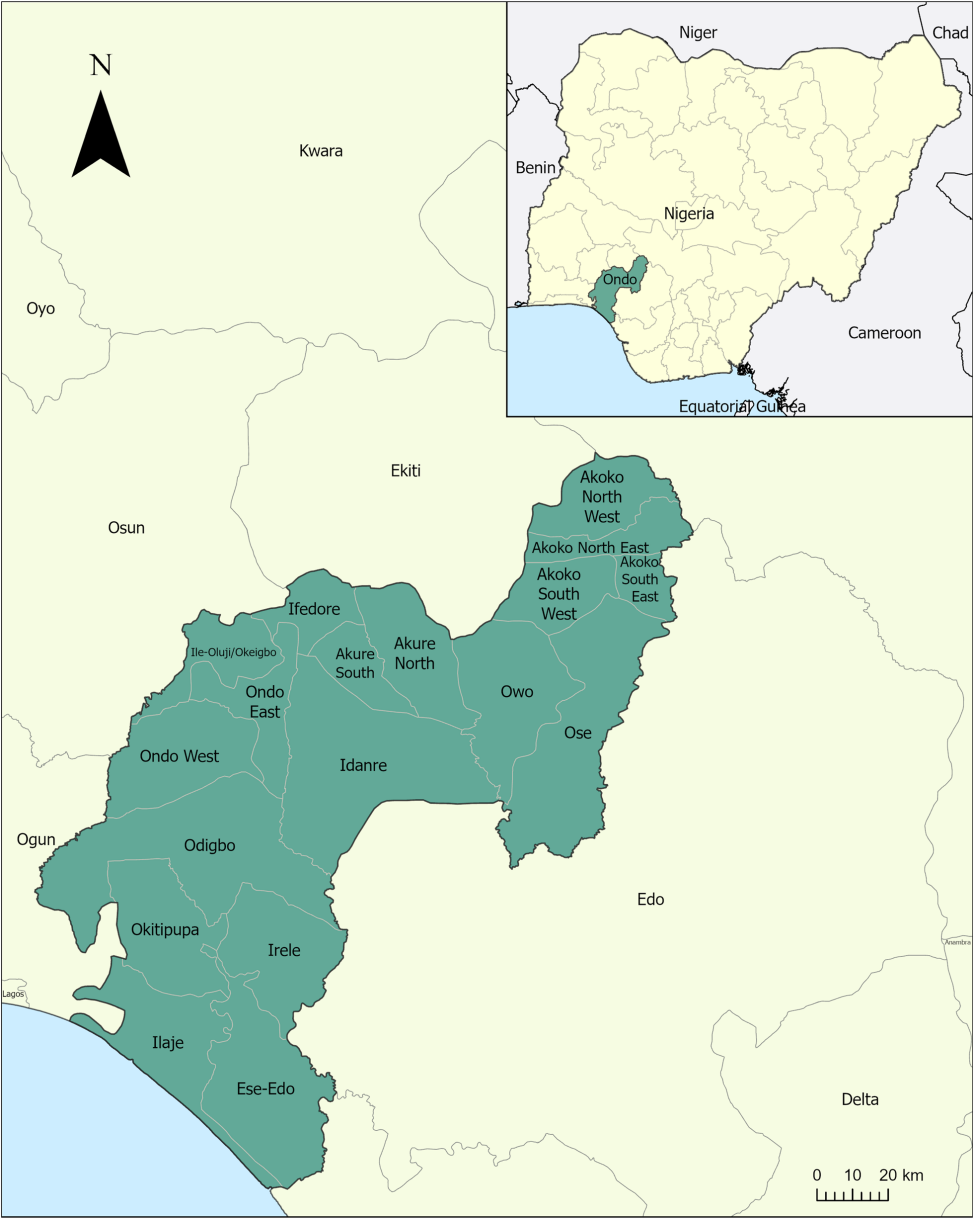
Location of Ondo state, Nigeria

Ondo state is situated in Southwest Nigeria. It covers a land area of approximately 15,000 km^2^ and is bordered by the states of Ekiti and Kogi to the north, Edo to the east, Delta to the southeast, and Osun and Ogun to the west and by the Bight of Benin in the Atlantic Ocean on the south as shown in Fig. 1.

Ondo state is made up of 18 Local Government Areas (LGAs) and 203 political wards. In 2021, the state population was projected to be approximately 5.3 million [8]. The 2018 Demographic and Health Survey found that Ondo state had a high malaria prevalence while access to ITNs was insufficient [9]. In 2018, 80% of households in Ondo state had at least one ITN, however only 55% of households had at least one ITN for every two people in the household, the recommended number by the WHO [9]. While 69% of the population had access to an ITN within their household, only 53% of the population slept under an ITN the night before the survey [9].

### ITN campaign process

#### Campaign planning and delivery

The ITN campaign was implemented by Malaria Consortium, in collaboration with the Nigerian National Malaria Elimination Programme (NMEP) and funded by Open Philanthropy and based on GiveWell’s recommendation.

#### Macro-quantification and procurement

To determine the number of ITNs required for the campaign, projected population of Ondo state was used based on Nigeria’s 2006 census [8]. To determine the number of ITNs required, the total population was divided by 1.8 and a 10% buffer was added as recommended by national and international guidelines [10, 11]. The resulting quantity was then rounded up to maximize the space available in shipping containers.

#### Micro-quantification and transportation

Micro-planning activities were concluded four months ahead of the campaign. The state team and LGA technical assistants were trained to conduct micro-planning activities, at LGA, ward, health facility and community levels, including micro-quantification of ITNs. LGA technical assistants then travelled to their respective LGAs to orientate the ward teams, carry out data collection and conduct advocacy visits. The orientation of ward teams, data collection and advocacy visits were carried out in a group setting with strict compliance with COVID-19 prevention protocols and guidelines. During the data collection the teams gathered ward (sketch) maps, community lists, the population of each ward, the location of distribution hubs and catchment areas, and the number of mobilisers and distributors required for each ward. The ward maps—developed by the ward focal person, officers in charge and ward development community representatives—have features including settlements, health facilities and distribution hubs.

#### Demand creation

Demand creation activities took place to raise awareness of, and support for, the ITN distribution campaign. Advocacy visits were carried out to state security services and security agencies, traditional and religious leaders and to government and ministry officials at state and LGA levels. Orientation sessions were also carried out with representatives of media organizations, town announcers and civil society organizations, who were tasked with increasing community awareness of the campaign and encouraging the uptake of ITNs. Orientation sessions included information about COVID-19, including the symptoms and preventive measures to reduce transmission risk. Communities were notified about the ITN campaign and the door-to-door delivery method through radio jingles, live media reporting, town announcements and a campaign flag-off ceremony.

#### Campaign strategy training

In November and December 2021 staff at state, LGA, and ward levels were trained to familiarize them with the COVID-19 protocols and adapted campaign strategy. Protocols to prevent COVID-19 transmission were introduced and monitored during all trainings. The adaptations included ensuring there was adequate space at training venues for participants to maintain a distance of at least 1.5 m apart, providing participants with face masks and ensuring hand sanitisers and hand washing facilities were available in and around the training venues.

#### Mobilization and distribution

Typically, ITN campaigns use a double-phase fixed-point distribution strategy. The first phase involves a mobilization visit to households. During the mobilization visit, enumeration teams collect information including the number of people living in the household and households are issued with a net card stating the number of ITNs they are eligible to collect at the distribution point. During the visit health education sessions are also carried out to provide information on malaria and how to use and care for nets. The mobilization and enumeration activities are typically carried out 1–2 weeks before the ITN distribution. The second phase is where representatives of households travel to a fixed location with their net cards to collect the ITNs they are eligible for. In comparison, this campaign adopted a single-phase door-to-door distribution strategy. This strategy involved a single visit to households where mobilization, enumeration and ITN distribution were carried out simultaneously. The single-phase approach aimed to avoid congregation of people at distribution hubs and thus reduce the number of contacts among the distribution teams and household members to prevent COVID-19 transmission. The door-to-door distribution approach involved the mobilization and distribution teams collecting ITNs from the distribution hub and delivering them ITNs directly to households instead of households collecting ITNs from a fixed point. Mobilization and distribution teams used different modes of transport to deliver ITNs—on foot, by bicycle, and by motorized transport—depending on the terrain and distance between households in each area. Following national guidelines for ITN distribution campaigns, one ITN was allocated for every two people in the household, with a maximum of four ITNs per household. Where there were an uneven number of household members the number was rounded up.

To adhere to the COVID-19 guidelines set by WHO and Nigeria’s National Centre for Disease Control, the campaign implemented the following steps during the distribution phase:

- Training distribution staff on COVID-19 and ways to prevent transmission;
- Providing distribution staff with personal protection equipment (PPE) including face masks and hand sanitisers;
- Maintaining COVID-19 prevention measures including physical distancing, consistent face mask use and hand hygiene practices; and
- Screening for symptoms of COVID-19 each day prior to distribution;

During the mobilization and distribution phase, each household received health education. This information was delivered in the local language and covered malaria, COVID-19 and how to use and care for ITNs.

#### Post-distribution survey

A household survey was conducted four months after the campaign in 52 sampled wards to monitor household ownership of ITNs.

#### Campaign digitisation

RedRose, a mobile data collection platform which includes geolocation functionality, was used to track attendance at training sessions and monitor cash and asset transfer throughout the mobilization and distribution activities. A second digital platform, SurveyCTO, was used for in-process and end-process monitoring.

#### Device set up

The information and communication technology (ICT) for Development (ICT4D) team were responsible for setting up the Android mobile phone devices in advance of the training and prior to the mobilization and distribution. The set up included checking whether devices worked, charging batteries and managing set up including geolocation settings.

#### Training on digital devices

A ICT4D team delivered a three-day training on technology deployment and operations for training and technology assistants to ensure teams were comfortable using the digital devices. The training covered use of the Android devices and mobile application and who to contact with issues. Hands-on practical simulations and role plays were used to promote effective learning.

LGA coordinators joined the final day of training to understand the roles and responsibilities of the training and technology assistants. This also allowed the LGA coordinators to meet the teams they would be supervising in advance to build rapport.

Cluster supervisors, who were responsible for supervising a collection of 2–3 wards, were also trained on the cash and asset transfer strategy. A cash and asset transfer strategy is used to monitor the movement of financial and material assets. For this campaign the cash and asset transfer strategy included monitoring personnel attendance at training sessions, and the payment of personnel and tracking the transfer of ITNs from the time of their delivery to the state warehouse to the receipt of the ITNs at the households. This campaign used Android mobile phones to enter training attendance data and ITN counts at storage facilities to improve tracking, accountability and efficiency during the campaign. Training for cluster supervisors included device syncing and data retrieval and distribution hub supervisors received practical sessions with the devices. During all training sessions COVID-19 transmission prevention protocols were followed. After training sessions, the devices were distributed to the mobilization and distribution teams.

#### Tracking attendance

All training sessions conducted at state, LGA and ward level were tracked using the mobile application. Each participant was given an identification (ID) badge which had a barcode to scan when arriving and leaving training sessions to track their attendance. Data were collected across all 66 training locations and captured information such as participant name, arrival and departure time and geocoordinates of the training location. The process of logging attendance was monitored by the ICT4D team and data were checked daily to ensure completeness.

#### Mobilization, enumeration and distribution of ITNs

The mobile application was also used during the mobilization, enumeration and distribution phase of the campaign. The mobilization and distribution teams used the application to capture household information—including the number of household members and the geolocation of the household—and to track the collection of ITNs from the distribution hub, delivery to households and return of excess nets to the distribution hubs at the end of each day. Household mobilizers, distributors, distribution hub supervisors and security officers were allocated ID cards to access the mobile application on the device. These ID cards allowed them to log the registration and issuance of ITNs to households and the collection and return of ITNs at the distribution hub. At the end of each day the mobilization and distribution teams returned to the distribution hub where data were checked by the distribution hub supervisor to verify the distribution data matched with the number of ITNs collected. The data were also reviewed by the state team to confirm the targeted number of ITNs had been distributed.

#### Monitoring and supervision

The ICT4D team supervised the digitization of the mobilization and distribution through two teams; the field monitoring team, who provided in-person assistance, and the help desk support team, who provided state-wide support through instant messaging and over the telephone. Training and Technology Assistants were also available at their respective ward clusters to provide additional supportive supervision. To ensure the devices were functioning correctly and the campaign was being carried out as planned, supervisors remotely tracked the digital devices using a mobile device management software.

In-process monitoring was also carried out by the state and LGA teams, LGA Technical Assistants and Campaign Implementation Teams using SurveyCTO. Each monitor randomly sampled ten households and three distribution hubs daily for the duration of the activity in any LGA or ward they visited.

### Data analysis

The data collected during the mobilization and distribution phase were monitored and analysed in real-time by the ICT4D team. Any inconsistent records were identified, and shared with the Campaign Implementation Team, LGA Coordinators, and Training and Technology Assistants for follow-up and resolution. At the end of each day the data were retrieved from the devices and entered in the daily ITN distribution summary sheet by the distribution hub supervisor. This enabled the team to monitor the success of the campaign by calculating the cumulative ITNs distributed and obtain the distribution rate. The undistributed ITNs returned by the mobilization and distribution team were also verified and recorded.

Daily review meetings were held at LG/A and state level to review the data collected and activities carried out. These meetings helped to identify and resolve problems quickly and improved the quality of implementation. At the end of the household mobilization and distribution the data were collated and signed off by the LGA managers and technical assistants and delivered to the State Malaria Elimination Programme Manager.

### Outcomes

#### Macro-quantification and procurement

The macro-quantification estimated the population of Ondo state to be 5,361,003 in 2021 (Table 1). The number of ITNs required including buffer was 3,276,169, so 3,300,000 ITNs were procured to cover the population. The micro-quantification recorded the state population as 5,298,162 and based on that the number of ITNs required including buffer was estimated to be 3,237,766 (i.e. 62,234 ITNs more than the number required after the micro-quantification).

**Table 1:** Results of the macro-quantification, micro-quantification and distribution

Indicators	Value
Population after macro-quantification	5,361,003
Households after macro-quantification	1,072,201
ITNs required after macro-quantification (including buffer)	2,978,335(3,276,169)
Population after micro-quantification	5,298,162
Households after micro-quantification	1,059,632
ITNs needed after micro-quantification (including buffer)	2,943,423 (3,237,766)
Population after household mobilization	5,623,729
Households after household mobilization	1,057,577
ITNs distributed	2,965,125

#### Micro-quantification and transportation

The data collected during the micro-quantification were amended after submission and the number of ITNs required was revised for some areas. This required ITNs to be redistributed from one LGA or ward to another to ensure there was sufficient stock to cover the change in population. In addition, recent data on communities and settlements in the state were not always available which resulted in some areas not being captured in the micro-quantification.

#### Mobilization and distribution

During the household mobilization and distribution 1,057,577 households were recorded and a total of 2,965,125 ITNs were distributed. The mobilization phase recorded 2055 fewer households and an increase of 325,567 in the population, compared to the microplanning results. The increase in population may be due to the microplanning using an estimate of five people per household. During the distribution 272,641 fewer ITNs were delivered compared to the number predicted by the microplanning. The difference between the number procured and the number distributed was 334,875, equivalent to 10.1% of the total ITNs procured. The post-distribution household survey, conducted four months after the campaign, found there was a significant increase in household ownership of nets. The percentage of households with at least one ITN increased from 23.3% to 79.8%, and the de facto population with access to an ITN within their households increased from 16.3% to 67.5%. The percentage of households with at least one campaign net for every two people was 53.5% overall (unpublished data). The post-campaign survey also showed that the number of nets received by households ranged from 0 to 10. Most households (79%) received between 1 and 4 campaign nets. Approximately 4% of households received 5 or more nets. Out of the sampled households, 17% did not receive campaign nets. No significant differences were observed among households with socio-economic status groups in terms of the mean number of nets received, indicating high level of equity of the campaign distribution.

### Lessons from the adapted campaign strategy

#### Benefits

The primary goal of adapting the distribution strategy was to ensure the ITN campaign could be implemented while adhering to COVID-19 restrictions. The single-phase and the door-to-door distribution approach was likely to have reduced the number of contacts between households and the distribution teams and mixing between residents from different households. In addition, a few other benefits were observed as a result of adopting a single-phase door-to-door approach. First, adopting this approach meant that enumeration, mobilisation and distribution was conducted simultaneously. Therefore, there was no need to distribute net cards to households in advance of the distribution. This was an advantage as it reduced the amount of paper required for the campaign and the cost of production and distribution of the cards. In addition, net cards can sometimes be lost between the enumeration and collection of ITNs, and this was not a problem due to the single-phase method. Second, the door-to-door approach was more convenient for households as they did not have to travel to a fixed location at a specific time to collect their ITNs. Finally, the door-to-door method meant that when distributing the ITNs information on how to hang and use a net could be tailored to suit each household.

#### Challenges

**Fig. 2:**
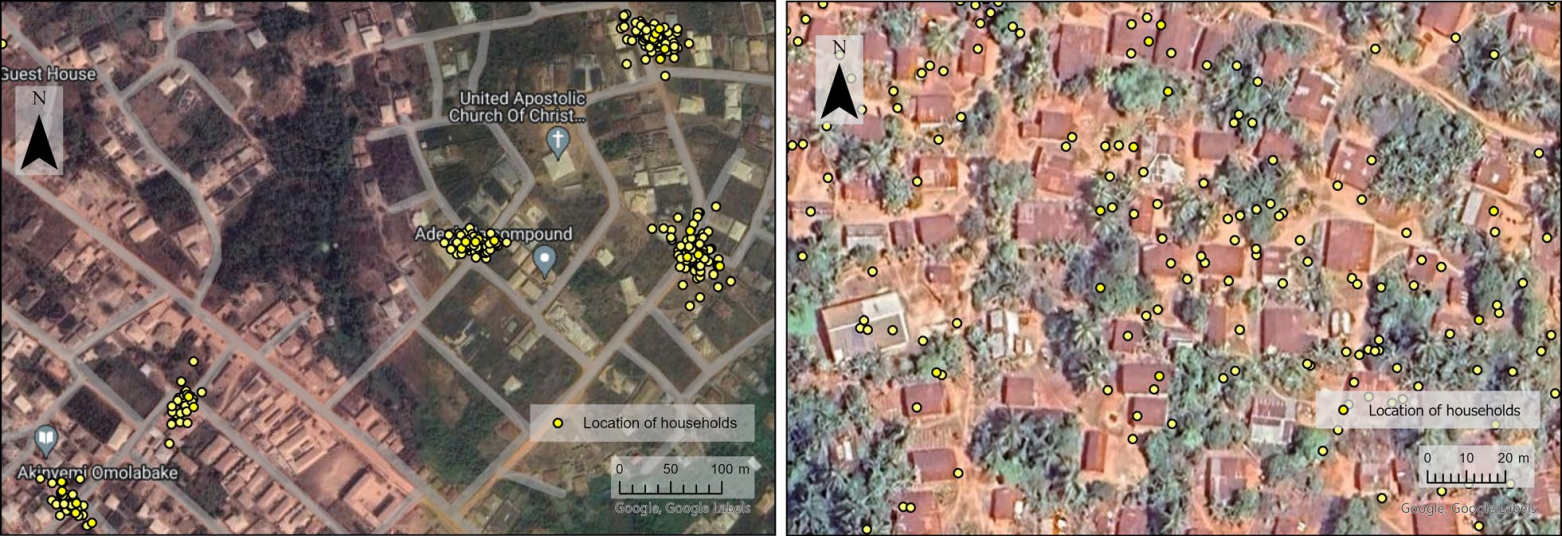
Data points demonstrating the geolocation of ITN distribution. In some areas the points demonstrate data points consistent with a door-to-door distribution strategy, in other areas there are clustering of data points which is more consistent with point distribution

Several challenges were also observed due to the adaptations made to the training schedule and mobilisation and distribution strategy. First, during the training, additional materials were developed to teach staff about COVID-19 symptoms and prevention. The agenda for training sessions was already full, and the extra information added pressure to this schedule. This led to insufficient time to focus on the main deliverables, which could have compromised the quality of delivery. In addition, using a single-phase approach meant household information was not collected in advance of the distribution. Normally there is time between the enumeration and distribution phase to ensure enough nets are at each distribution hub and make adjustments where needed. For this reason, there was a greater reliance on the micro-quantification for calculating the number of ITNs needed in each area. In addition, the door-to-door approach was more challenging for distribution teams requiring more time and effort to travel from house to house with the ITNs. The ITNs are heavy and carrying them to each household was tiring for the teams and in some areas, where the houses are far apart, the distribution teams had to pay for transport out of their own pocket. In addition, if no one was home at the time of the visit the distribution teams had to re-visit at another time. In some cases, distribution teams did not re-visit and these households were missed. The additional time needed to distribute nets had a knock-on effect and reduced the amount of time for health talks. The monitoring teams found in some areas the quality of the health talks was insufficient and resulted in household not airing and hanging their nets in good time. Finally, households were notified that the distributors would deliver ITNs to their door. However, in a few areas the door-to-door guidance was not followed and distribution teams delivered ITNs using a fixed-point distribution instead of visiting each household (Fig. 2). There were a variety of reasons why a point method was used including security concerns, a perceived lack of ITNs for all households and difficulties transporting ITNs. This caused confusion as households had waited at home for ITNs to be delivered and in some cases, households did not receive or collect ITNs.

### Lessons from digitizing the campaign

#### Benefits

**Fig. 3:**
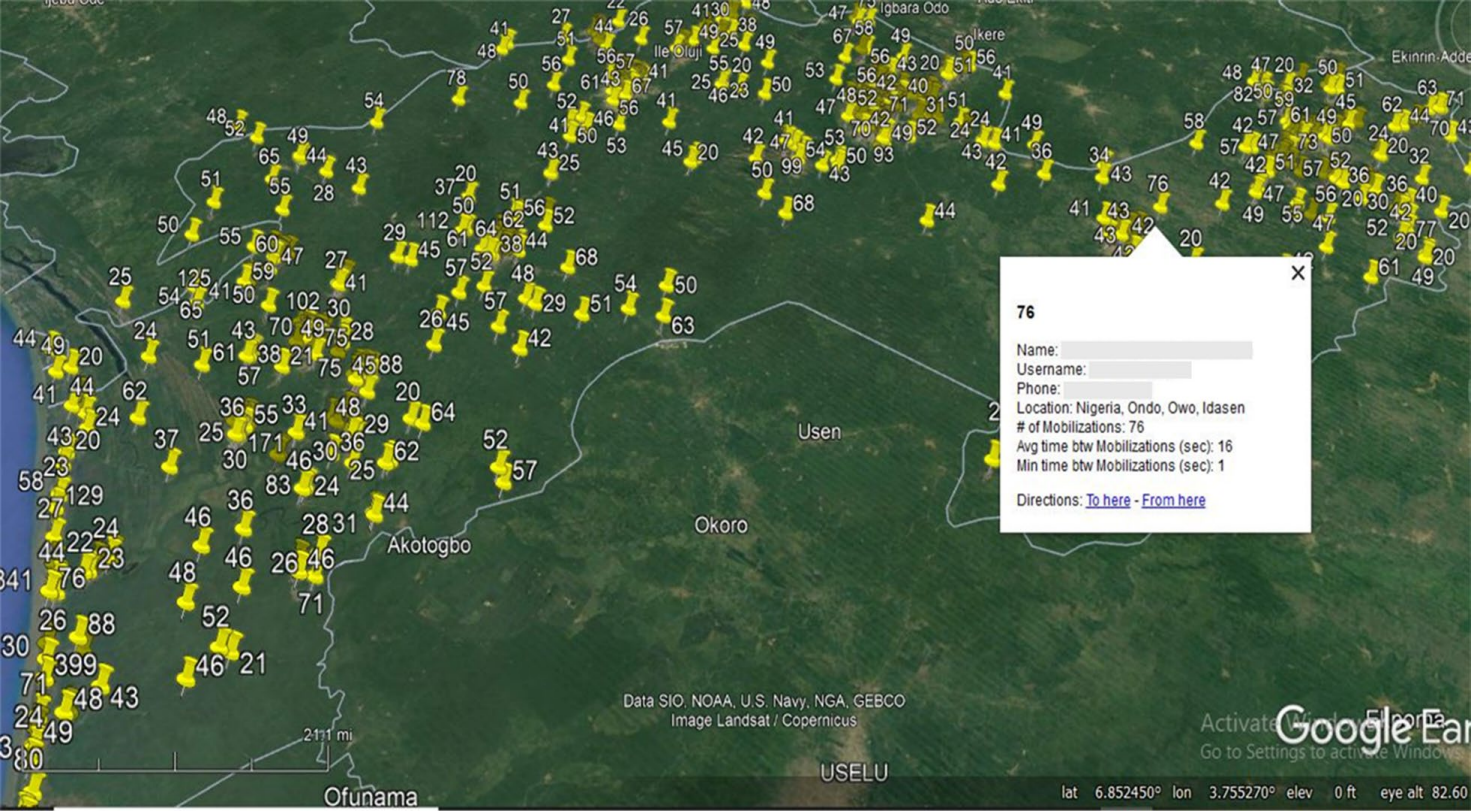
Map showing mobilization and distribution with each mobilizers information

Digitising the ITN campaign had several benefits. Collecting data using digital devices meant teams did not have to carry paper forms around with them which can be heavy. In addition, collecting data digitally allowed supervisors to carry out in-process monitoring of the campaign in real-time. Supervisors were able to track the location of each device to ensure teams were carrying out the campaign in their assigned areas. The devices also recorded information such as the number of distributions carried out by the team and the length of time between distributions (Fig. 3). Being able to track the devices meant supervisors could monitor the functionality of the devices and the ICT4D team could provide support remotely or in person to rectify any issues quickly. Furthermore, tracking the location of the distribution teams was important for ensuring the safety of the teams and their devices and enabled the ICT4D teams to locate them if deployed to support. In addition, the mobile application was used for stock taking at distribution hubs and being able to view and analyse these data in real-time meant the supervision teams could identify if stock was low and make arrangements to transfer ITNs from other distribution hubs before stock ran out. Furthermore, recording the geolocation for each distribution meant that the data could be plotted on a map to view coverage across the state and identify any areas that had been missed. Finally, collecting data on digital devices meant data could be transferred remotely which reduce contact between distribution teams and supervisors which was an important consideration for delivering the campaign under COVID-19 restrictions.

#### Challenges

During the ITN distribution campaign in Ondo state a few challenges were identified. First, when logging distribution data in the RedRose app was when distributors did not have the location settings set to a precise enough value. This resulted in some distributors being recorded in inaccurate locations and on occasion the data were geocoded to locations in a different state especially in border areas. However, the use of the RedRose app led to this error being identified quickly and the importance of the accuracy of this setting could be reiterated to the team while the distribution was still ongoing.

In addition, there were challenges when logging the collection and return of ITNs at distribution hubs. Ideally all distributors would have scanned their ID badge to record the number collected or returned. However, in practice distributors were entering their information manually which resulted in data entry errors and ITNs being logged against an incorrect distribution hub. This error resulted in discrepancies between predicted stock and actual stock of ITNs at the distribution. In some cases, distributors did not receive ID badges in time due to personnel lists arriving late from the state and then being updated prior to and during the campaign.

## Discussion

The adaptations made to the ITN distribution in Ondo state in 2021 ensured the campaign could be implemented during the COVID-19 pandemic by putting in place safety measures to prevent transmission of the virus. If the campaign had not been implemented households may have been left without adequate malaria prevention methods and the consequences could have been severe [12]. However, the inclusion of information on COVID-19 in the training sessions put pressure on an already full training schedule, risking participant fatigue and less focus being paid to important operational information. To improve the training schedule in the future, trainers should identify the critical information needed by each group of participants to perform their role. Critical information should be presented first with introductory information, for example outlining malaria epidemiology, provided later in the programme. Alternatively, some information could be delivered in-person with optional material delivered through self-study either in paper-based or digital formats. Although online training was insufficient for training staff to carry out activities during ITN campaigns in Uganda [13] and a pilot study in Nigeria has shown online modules can be an effective way to deliver foundational health information [14]. Adopting a blended-learning approach by providing foundational information through a self-paced online platform and information specific to the campaign through in-person training could help to cover all the necessary content while reducing information overload. Further study is needed to confirm whether this approach would be feasible, acceptable and beneficial.

One significant challenge present during the planning of this campaign was the absence of up-to-date population estimates for national and sub-national levels. The macroplanning for this campaign used data from a census conducted 15 years prior [8]. High-quality precise data is essential to procure accurate resources. Improvements in data quality could lead to savings in purchasing commodities and transportation costs while avoiding under-estimations.

In addition, poor data quality has implications for the allocation of resources. Some of the information gathered for the microplanning missed settlements or had to be updated. Remote sensing products have been used to map settlements of mobile populations in Myanmar and may be a useful tool to ensure settlements not identified by community lists are allocated resources [15]. Mobile phone data has also been used to track population movement which may be useful for identifying population changes between microplanning and distribution if using a single-phase approach [16].

Issues with data quality for planning and allocation are not unique to ITN campaigns and is a factor affecting the delivery of many large-scale health programmes [17]. In fact, the need for improvements in reliable and timely data was highlighted in the 2021 update of the Global Technical Strategy for Malaria 2016–2030 [18] and given the increased cost of newer types of ITNs [19] and shortfall in international funding streams such as the Global Fund [20] it is increasingly important to use resources efficiently. In addition, automated systems could be used to notify programme planners when adjustments are needed.

The single-phase door-to-door approach was more convenient for households, removing the necessity to travel to a fixed point, at a specified time. In addition, the removal of net cards has the potential to reduce the gap between allocation and collection and improve ITN coverage. However, the door-to-door approach was more time and labour-intensive for health workers, who often had to carry a large number of nets long distances between houses. More investigation is needed to understand if the adapted approach improved household ITN coverage compared to the fixed-point approach. If the results are positive these will need to be considered in combination with the increased burden on health workers, and strategies may be needed to facilitate door-to-door distribution, for example providing health workers with transportation.

An additional consideration is the impact of the door-to-door approach on the quality of health education delivered to households. Although point distribution does not allow information to be tailored to each household, it is easier to maintain the quality of information given and reduces disparity between households. To maintain the quality of health education delivered as part of a door-to-door approach, key information could be standardized and provided through locally appropriate channels including pre-recorded audio or video messages. Studies have shown health education videos are an effective way to deliver information to communities [21, 22] and a pilot study in South Africa demonstrated feasibility and acceptability when delivered by community health workers [23].

Finally, digitizing the campaign to enable door-to-door distribution of ITNs had several benefits that ensured high-quality implementation while reducing the risk of COVID-19 transmission between households and mobilisation and distribution teams. The use of real-time monitoring, evaluation and response allowed improvements to be made during the campaign and the geolocation data allowed supervisors to identify where door-to-door distribution was challenging and any areas that were missed. The information collected during this campaign will also help to inform and plan future ITN distributions. In future, adjustments can be made to optimize the digitisation, for example, implementing data validation rules to reduce data entry errors, preventing geolocation settings from being changed or disabled and ringfencing data to a geographical area. In addition, more guidance could be given to mobilization and distribution team about the importance of accurate data entry and maintaining original device set up to avoid errors.

## Conclusion

The adaptations made to the delivery strategy in Ondo state allowed the ITN campaign to be delivered during the COVID-19 pandemic and ensured the safety of staff and communities. The changes to the mobilization and distribution of the campaign increased the workload for distribution teams but reduced some of the barriers that may discourage households from collecting nets. Digital tools are a helpful way to improve the planning and delivery of ITN campaigns efficiently. However, the use of these tools requires additional training for mobilisation and distribution teams to ensure these tools are used correctly. To further optimize the targeting and delivery of ITN campaigns high-quality population estimates are needed to avoid over- and under-estimation of required resources.

## Data Availability

No datasets were generated or analysed during the current study.

## Abbreviations

ICT: Information communication technology
ICT4D: Information communication technology for development
ID: Identification
ITN: Insecticide-treated nets
LGA: Local government area
NMEP: National Malaria Elimination Programme
PPE: Personal protective equipment
WHO: World Health Organization
